# Metformin-Associated Gastrointestinal Adverse Events Are Reduced by Probiotics: A Meta-Analysis

**DOI:** 10.3390/ph17070898

**Published:** 2024-07-05

**Authors:** Izabela Szymczak-Pajor, Józef Drzewoski, Sylwia Wenclewska, Agnieszka Śliwińska

**Affiliations:** 1 Department of Nucleic Acid Biochemistry, Medical University of Lodz, 251 Pomorska Str., 92-213 Lodz, Poland; 2 Central Teaching Hospital of the Medical University of Lodz, 251 Pomorska Str., 92-213 Lodz, Poland; jozef.drzewoski@umed.lodz.pl; 3 Provincial Hospital Named after Primate Cardinal Stefan Wyszyński, 7 Armii Krajowej Str., 98-200 Sieradz, Poland; sylwiawenclewska@wp.pl

**Keywords:** metformin, oral glucose-lowering drugs (GLDs), probiotics, gastrointestinal (GI) adverse events

## Abstract

Metformin, one of the most frequently used oral glucose-lowering drugs (GLDs), is associated with the occurrence of gastrointestinal (GI) adverse events in approximately 20% of users. These unwanted actions result in non-compliance or even discontinuation of metformin therapy. The aim of the presented meta-analysis was to determine whether adding a drug from the group of sulfonylureas, glitazones, DPP-IV inhibitors, or probiotics to metformin monotherapy may affect the risk of GI side effects. The material for this meta-analysis comprised data from 26 randomized controlled clinical trials (RCTs) published in English. This meta-analysis included 41,048 patients. The PubMed, Cochrane Library, and Clinical Trials databases were thoroughly searched to find relevant RCTs. The Population, Intervention, Comparison, Outcomes, and Study Type (PICOT) structure was used to formulate study selection criteria and the research question. Cochrane Review Manager Software 5.4 was used to carry out analysis of collected data. The results were presented as relative risk (RR) and 95% confidence interval (95% CI) for each group, and *p* < 0.05 was considered as statistically significant. As expected from clinical practice, metformin was associated with a markedly increased risk of abdominal pain, nausea, and vomiting compared to placebo. In comparison to other GLDs, taking metformin was related to an elevated risk of diarrhea and abdominal pain and to a lowered risk of vomiting and bloating. In turn, adding other GLDs to metformin treatment was associated with an elevated risk of nausea and vomiting than treatment with metformin in monotherapy. However, adding probiotics to metformin therapy was related to a decreased risk of diarrhea, bloating, and constipation. The obtained results demonstrate that the combination of metformin with other GLDs may elevate the risk of nausea and vomiting, whereas combination with probiotics decreases the risk of diarrhea, bloating, and constipation. Thus, the results of our meta-analysis suggest that probiotics may reduce the risk of some GI side effects in people with type 2 diabetes mellitus (T2DM) who started treatment with metformin.

## 1. Introduction

Metformin, a dimethylbiguanide derived from *Gallega officinalis*, is an oral glucose-lowering drug [1]. The anti-hyperglycemic effect of this drug is based primarily on the inhibition of hepatic gluconeogenesis, which contributes to the reduction in glucose secretion by the liver. Metformin also increases the liver’s sensitivity to insulin and switches hepatocytes from the anabolic pathway associated with gluconeogenesis to the catabolic pathway related to glycolysis, which in turn leads to reduced energy consumption. The drug also increases insulin sensitivity in peripheral tissues, which contributes to increased glucose uptake and utilization by skeletal muscle and adipose tissue [2,3]. It is worth emphasizing that both European and American guidelines have been recommending the use of metformin as the first-line drug in the treatment of type 2 diabetes (T2DM). Recently, the American Diabetes Association (ADA) and European Association for the Study of Diabetes (EASD) issued a recommendation that the decision regarding the choice of drug to initiate treatment should also take into account comorbidities. This recommendation is based on the domain of patient-centered pharmacotherapy and allows treatment with glucagon like peptide 1 receptor agonists (GLP-1 RA) and sodium glucose cotransporter type 2 inhibitors (SGLT-2i) to be initiated despite the use of metformin in patients with risk or existing renal and/or cardiovascular disease [4]. Nevertheless, metformin is still a safe, cheap, and very widely used drug with a minimal risk of hypoglycemia and impact on body weight changes [5].

There is abundant evidence that the metformin-related glucose-lowering effect also occurs through the intestine [6]. Enterocytes are the first cells exposed to metformin. Metformin not only increases glucose uptake from the intestinal lumen via GLUT-2 relocation but also increases intestinal glucose utilization by producing lactate. In turn, lactate is used to produce glucose in the liver [3]. The intestine can also be considered as the main site of adverse events associated with metformin treatment [7]. Evidence shows that many patients treated with metformin develop intolerance due to gastrointestinal (GI) adverse events, mainly diarrhea, nausea vomiting, abdominal pain, and bloating, which affect up to 20% of patients. In some cases, these adverse events lead to poorer adherence, treatment discontinuation, and poorer health-associated quality of life [8,9,10,11,12,13]. The mechanism(s) responsible for metformin intolerance are not fully understood. The proposed hypotheses include the accumulation of histamine [14], serotonin [15], bile acids [16], increased lactate production [17] and some genetic factors associated with polymorphism of the organic cation transporter 1 (OCT1) gene [18,19]. There are several potential suggestions explaining the relationship between the occurrence of GI adverse events and metformin treatment. Firstly, metformin shows serotonergic-like effect due to certain structural similarities with agonists of the 5-HT3 receptor. Intestinal release of 5-HT (serotonin) may contribute to occurrence of GI symptoms, i.e., nausea, diarrhea, and vomiting [15,20]. The second potential hypothesis highlighted that genetic variations in OCT1 may be related to the absorption of metformin from the intestinal lumen, whereby decreased transport by this transporter may elevate metformin concentrations in the intestine in prone individuals contributing to the elevation of GI adverse events [18,19]. Metformin also reduces absorption of bile acids, leading to osmotic diarrhea [16]. The effect of metformin and oral glucose-lowering drugs (GLDs) on the microbiota is the third mechanism that may influence the frequency of GI side effects.

As suggested by Bryrup et al., metformin may change the composition of the intestinal microflora in men with normal glucose metabolism. Based on this observation, it is likely that the type of bacteria found in the digestive tract increases the risk of GI side effects from metformin [21]. It is believed that both undesirable side effects and therapeutic effects of metformin may be related to changes in the intestinal microflora [22]. The results of a systematic review of clinical trials and observational studies carried out by Petahk et al. have identified that metformin increased the abundance of *Blautia*, *Butyrivibrio*, *Escherichia*, *Biophila*, and *Bifidobacterium* whereas decreased the abundance of *Intestinibacter*, *Clostridium*, *Lactobacillus*, *Bacillus*, and *Alistipes* [23]. The composition of the physiological human intestinal microbiota includes *Actinobacteria*, *Bacteroidetes*, *Firmiciutes*, *Lactobacillae*, *Streptococci,* and *Enterobacteria* [24]. In turn, T2DM patients show an increase in multiple pathogenic bacteria, i.e., *Clostridium hathewayi*, *Clostridium symbiosum*, *Escherichia coli*, *Bacteroides vulgatus*, *Veillonella denticariosi,* and *Lactobacillus* whereas a decrease in *Clostridium coccoides*, *Clostridium leptum*, *Akkermansia muciniphila*. *Faecolibacterium prausnitzii*, *Clostridium*, and *Fusobacterium* [25]. The effect of metformin on biodiversity of intestinal microflora may have an impact on metabolism in patients with T2DM via the regulation of intestinal glucose uptake and glucose homeostasis, promoting short-chain fatty acid (SCFA)-producing bacteria, enhancing the gut-related peptide secretion, i.e., glucagon-like peptode-1, and regulating the bile acid turnover [26,27]. In turn, the extended-release formulations of metformin or gradually increasing the initial dosage of the drug may contribute to GI metformin intolerance relief is very limited [17]. In practice, to minimize the GI adverse events, a decrease in the dose of metformin or ceasing it should be considered [28]. In as many as 5% of people taking metformin, the severity of GI adverse effects contributes to the discontinuation of treatment [10]. There are other antidiabetic drugs that could be taken in the case of metformin intolerance such as sulfonylurea derivatives, although they may cause hypoglycemia, which is particularly dangerous in older people. Although, the newer GLDs do not cause hypoglycemia, they are expensive and unobtainable, and some of them (mainly GLP-1 receptor agonists) can cause serious GI adverse effects that require the discontinuation of their use [29]. The premises based on high effectiveness, safety, and widespread availability of cheap generics emphasize the leading position of metformin in the treatment of T2DM [30]. Therefore, a scientifically interesting and important clinical issue is whether administering other GLDs or probiotics with metformin may affect the incidence of GI adverse events, since other GLDs and probiotics act on intestinal microbiome [31]. Therefore, the aim of our meta-analysis was to evaluate whether adding GLDs or probiotics to metformin therapy affects the risk of GI side effects.

## 2. Results

### 2.1. The Studies Included into Meta-Analysis

**Figure 1:**
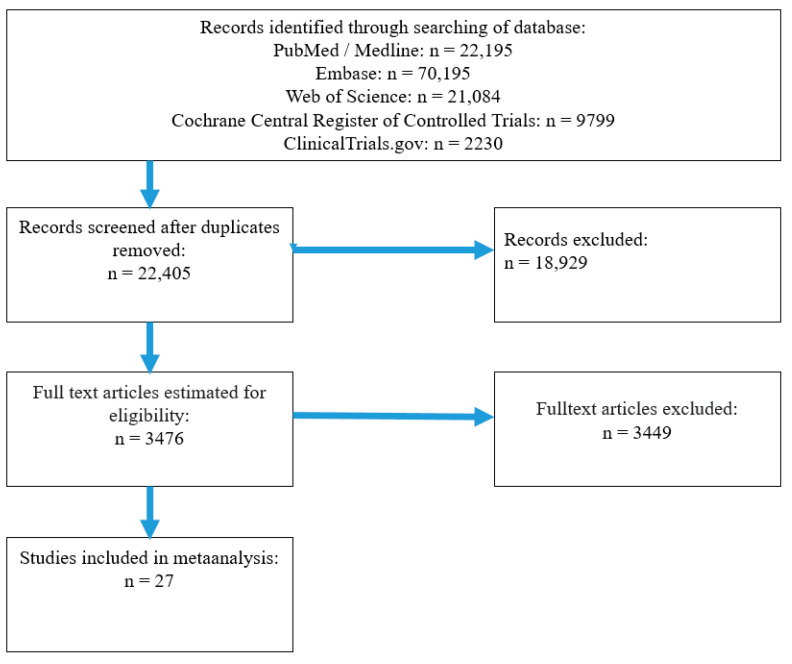
Flowchart of screening procedure.

As presented in Figure 1, database screening identified 125,203 associated articles. In total, 102,798 duplicates were excluded. After quality evaluation and full-text screening carried out by our researchers, 27 articles meet the inclusion criteria.

### 2.2. Assessment of Methodological Quality—Risk of Bias

**Figure 2:**
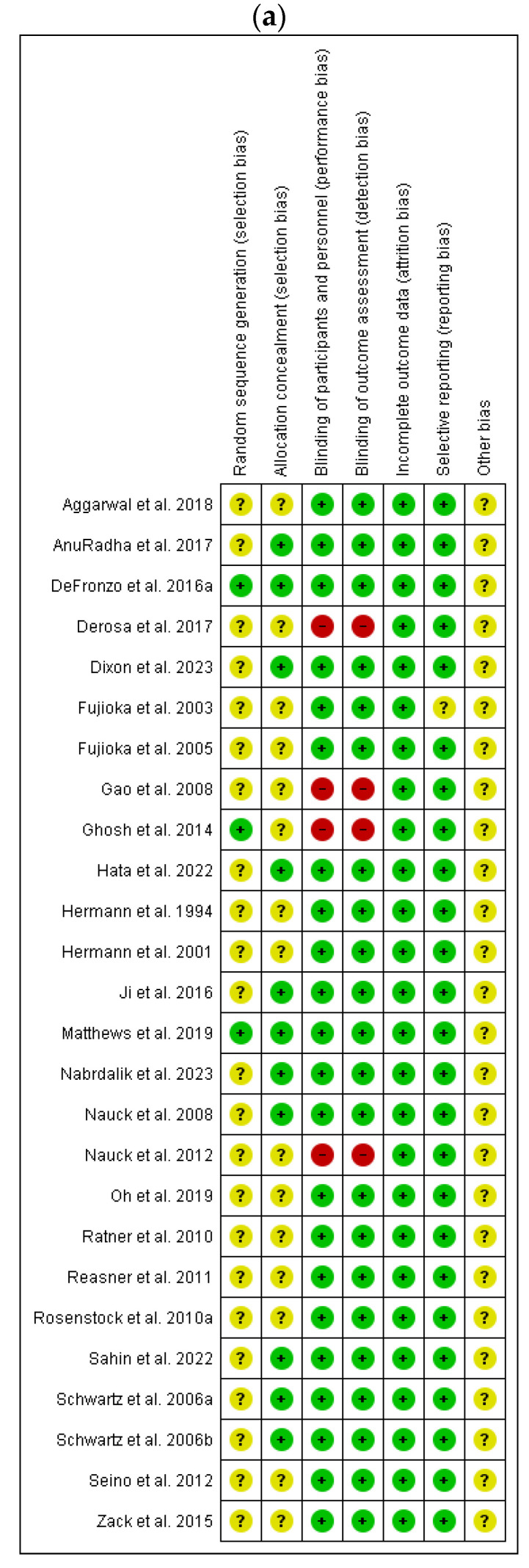

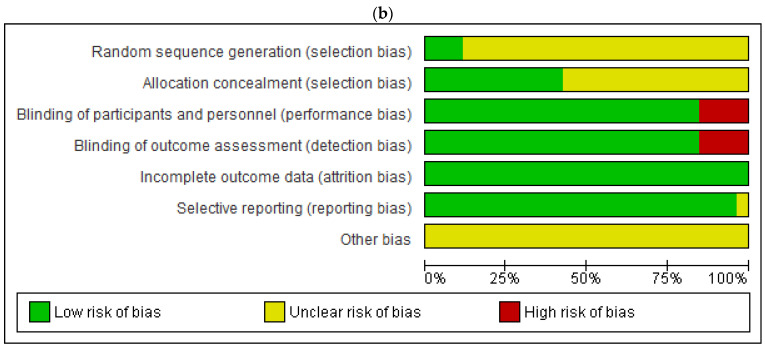
(**a**) Risk of bias summary: review authors’ judgements about each risk of bias item for each included study. (**b**) Risk of bias graph: review authors’ judgements about each risk of bias item presented as percentages across all included studies [32,33,34,35,36,37,38,39,40,41,42,43,44,45,46,47,48,49,50,51,52,53,54,55,56,57].

A total of 27 studies were included in the estimation of risk of bias. During risk of bias assessment, the following items were assessed: item 1: random sequence generation (selection bias); item 2: allocation concealment (selection bias); item 3: blinding of participants and personnel (performance bias); item 4: blinding of outcome assessment (detection bias); item 5: incomplete outcome data (attrition bias); item 6: selective reporting (reporting bias); and 7 item: other bias. Evaluated studies had a low risk of bias in estimated risk of bias items. Only four of the assessed studies had a high risk of bias in three and four items. All randomized controlled clinical trials (TRCs) included into the presented meta-analysis adhered to high standards; thus, the evaluated risk of bias was relatively low. The results of methodological evaluation of risk of bias are presented in Figure 2.

### 2.3. The Use of Metformin Increases the Risk of Abdominal Pain, Nausea, and Vomiting Relative to Placebo

**Figure 3:**
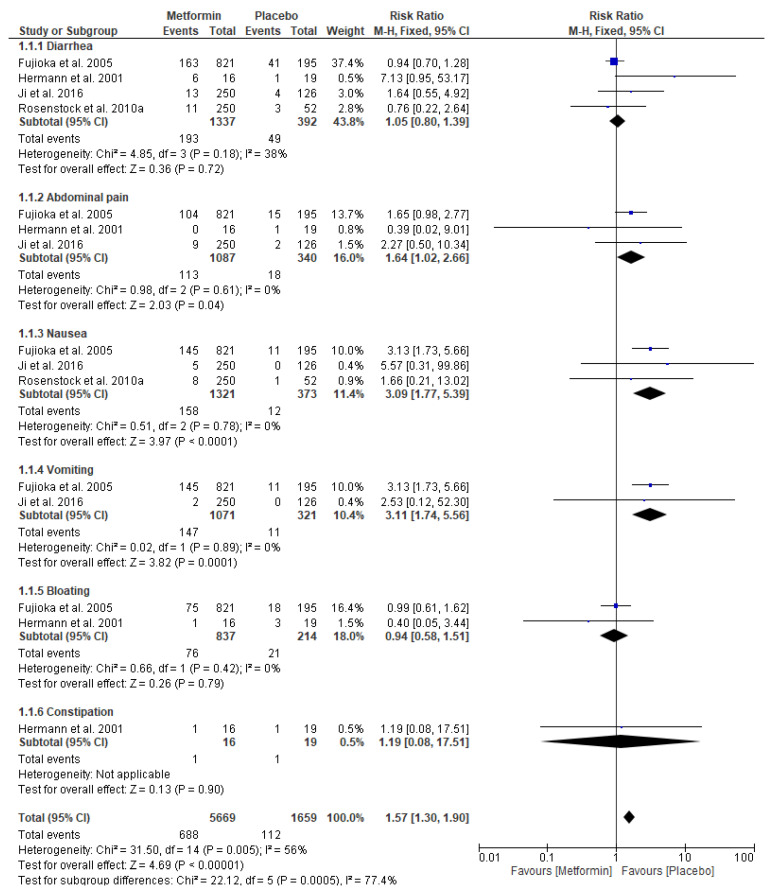
Risk ratio for gastrointestinal (GI) adverse events in patients taking metformin (intervention) vs. placebo (comparator) [37,43,44,52].

Figure 3 presents a forest plot for the risk ratio of GI adverse events in patients taking metformin (intervention) vs. placebo (comparator). For metformin vs. placebo, four original papers were eligible for the meta-analysis. The pooled data included 5669 patients in the metformin intervention group and 1659 patients in the placebo group. For the analysis in subgroups based on the type of GI adverse events, the meta-analysis showed a significantly increased risk of abdominal pain (risk ratio (RR) = 1.64; 95% confidence interval [CI]: [1.02, 2.66], *p* = 0.04), a significantly increased risk of nausea (RR = 3.09; 95% confidence interval [CI]: [1.77, 5.39], *p* < 0.0001), and a markedly elevated risk of vomiting (RR = 3.11; 95% confidence interval [CI]: [1.74, 5.56], *p* = 0.0001) in patients taking metformin compared to placebo. We did not find any significant risks of diarrhea (RR = 1.05; 95% confidence interval [CI]: [0.80, 1.39], *p* = 0.72), bloating (RR = 0.94; 95% confidence interval [CI]: [0.58, 1.51], *p* = 0.79), or constipation (RR = 1.19; 95% confidence interval [CI]: [0.08, 17.51], *p* = 0.90) in the intervention group vs. placebo.

**Figure 4:**
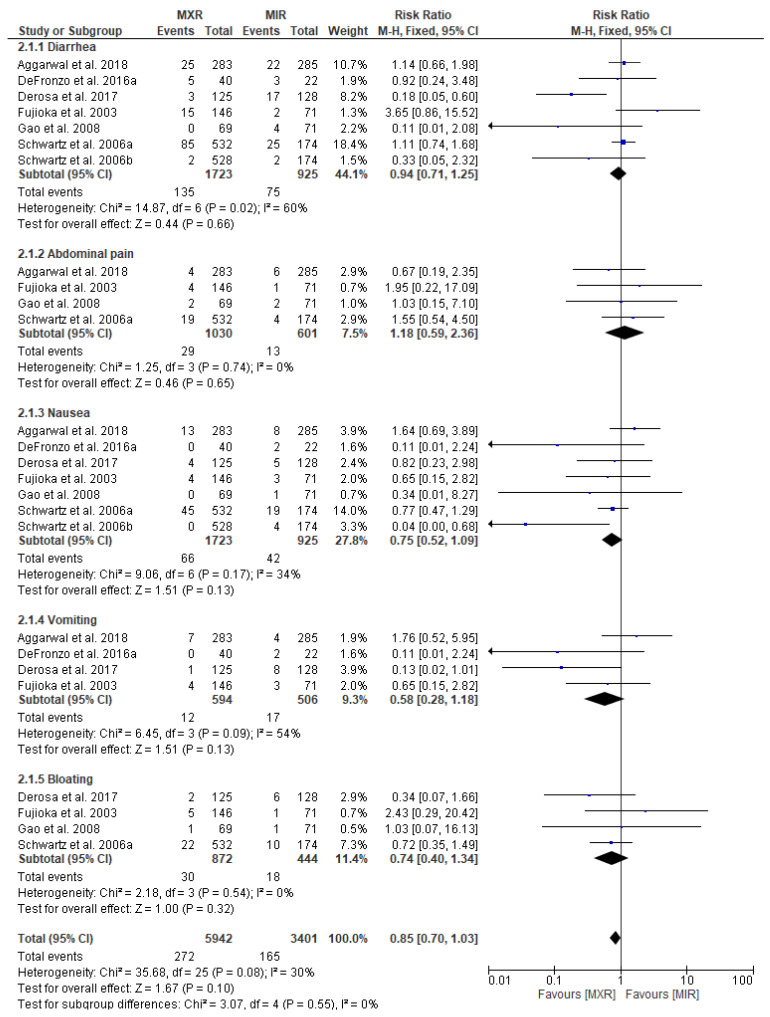
Risk ratio for GI adverse events in patients taking metformin extended release (MXR (intervention)) vs. metformin immediate release (MIR (comparator)) [32,34,35,38,39,54,55].

We also evaluated whether the risk of GI adverse events differs between patients taking metformin extended release (MXR) (MXR (intervention)) in relation to patients taking metformin immediate release (MIR) (MIR (comparator)), as shown in Figure 4. For MXR vs. MIR 7, original papers were eligible for meta-analysis. Pooled data included 5942 patients in the MXR intervention group and 3401 patients in the MIR comparator group. We did not observe any significant risks of diarrhea (RR = 0.94; 95% confidence interval [CI]: [0.71, 1.25], *p* = 0.66), abdominal pain (RR = 1.18; 95% confidence interval [CI]: [0.59, 2.36], *p* = 0.65), nausea (RR = 0.75; 95% confidence interval [CI]: [0.52, 1.09], *p* = 0.13), vomiting (RR = 0.58; 95% confidence interval [CI]: [0.28, 1.18], *p* = 0.13), and bloating (RR = 0.74; 95% confidence interval [CI]: [0.40, 1.34], *p* = 0.32) between the MXR group vs. the MIR group. Thus, the form of metformin does not affect the risk of GI adverse events.

### 2.4. The Use of Metformin Is Associated with an Increased Risk of Diarrhea and Abdominal Pain, Whereas Other Oral Glucose-Lowering Drugs (GLDs) Elevate the Risk of Vomiting and Bloating

**Figure 5:**
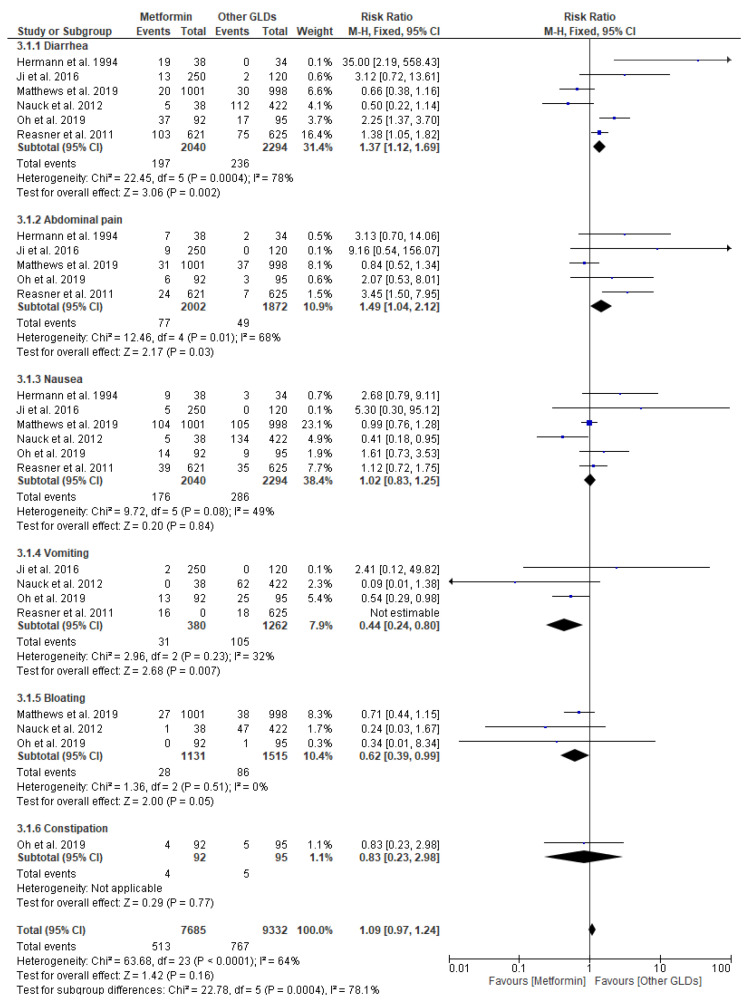
Risk ratio for GI adverse events in patients taking metformin (intervention) vs. other oral glucose-lowering drugs (GLDs (comparator)) [42,44,45,47,49,51].

The risk of GI adverse events in patients taking metformin (intervention) in comparison to other (GLDs (comparator)) is depicted in Figure 5. For metformin vs. other GLDs, six original papers were eligible for the meta-analysis. Pooled data included 7685 patients in the metformin intervention group and 9332 patients in the GLD group. For the analysis in subgroups based on the type of GI adverse events, the results of meta-analysis showed a pronouncedly increased risk of diarrhea (RR = 1.37; 95% confidence interval [CI]: [1.12, 1.69], *p* = 0.002) and abdominal pain (RR = 1.49; 95% confidence interval [CI]: [1.04, 2.12], *p* = 0.03) in the group of patients taking metformin in relation to other GLDs. Our meta-analysis also showed a markedly decreased risk of vomiting (RR = 0.44; 95% confidence interval [CI]: [0.24, 0.80], *p* = 0.007) and bloating (RR = 0.62; 95% confidence interval [CI]: [0.39, 0.99], *p* = 0.05) in patients taking metformin compared to GLDs. We did not observe any significant risks of nausea (RR = 1.02; 95% confidence interval [CI]: [0.83, 1.25], *p* = 0.84) and constipation (RR = 0.83; 95% confidence interval [CI]: [0.23, 2.98], *p* = 0.77) in the metformin group vs. other GLD groups. Thus, the treatment with oral hypoglycemic drugs, such as metformin, sulfonylurea derivatives, glitazones, and DPP-IV inhibitors, is associated with a higher risk of GI adverse events.

### 2.5. Adding Other GLDs to Metformin Increases the Risk of Nausea and Vomiting as Compared to the Use of Metformin Monotherapy

**Figure 6:**
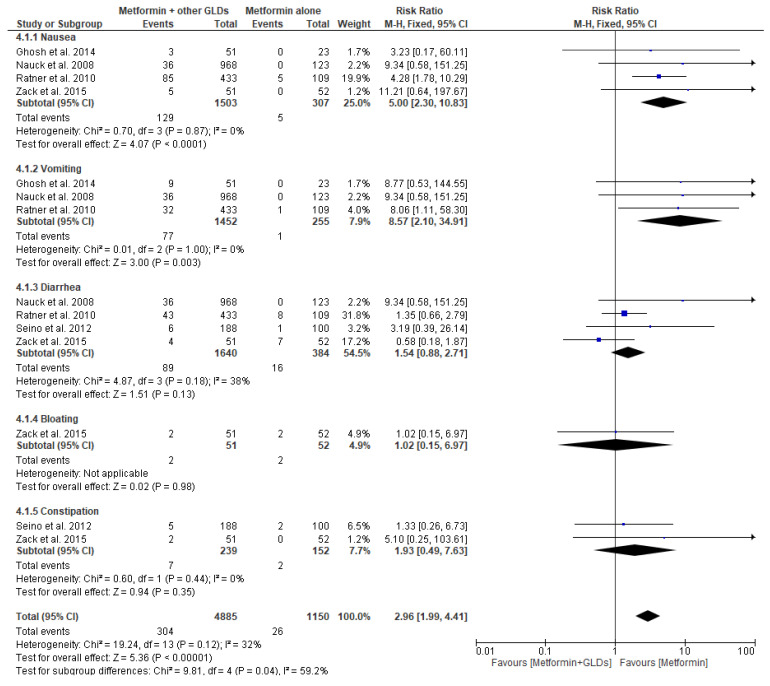
Risk ratio for GI adverse events in patients taking metformin + other oral glucose-lowering drugs (GLDs) (intervention) vs. metformin monotherapy (comparator) [38,40,50,56,57].

Figure 6 presents a forest plot for risk ratio for GI adverse events in patients taking metformin with other GLDs (intervention) in comparison to metformin monotherapy (comparator). For metformin with other GLDs vs. metformin alone, five original papers were eligible for the meta-analysis. Pooled data included 4885 patients in the metformin with other GLDs intervention group and 1150 patients in the metformin alone group. For the analysis in subgroups based on the type of GI adverse events, the meta-analysis showed a pronouncedly increased risk of nausea (RR = 5.00; 95% confidence interval [CI]: [2.30, 10.83], *p* < 0.0001) and vomiting (RR = 8.57; 95% confidence interval [CI]: [2.10, 34.91], *p* = 0.003) in the group of patients taking metformin and GLDs in relation to patients taking metformin alone. We did not observe any significant risks of diarrhea (RR = 1.54; 95% confidence interval [CI]: [0.88, 2.71], *p* = 0.13), bloating (RR = 1.02; 95% confidence interval [CI]: [0.15, 6.97], *p* = 0.77), and constipation (RR = 1.93; 95% confidence interval [CI]: [0.49, 7.63], *p* = 0.35) between the metformin and other GLD group vs. metformin alone group. To conclude, our data suggest that co-administering sulfonylurea derivatives, glitazones, or DPP-IV inhibitors with metformin therapy additionally increases the risk of GI adverse events such as nausea and vomiting.

### 2.6. Adding Probiotics to Metformin Compared to Metformin Monotherapy Decreases the Risk of Diarrhea, Bloating, and Constipation

**Figure 7:**
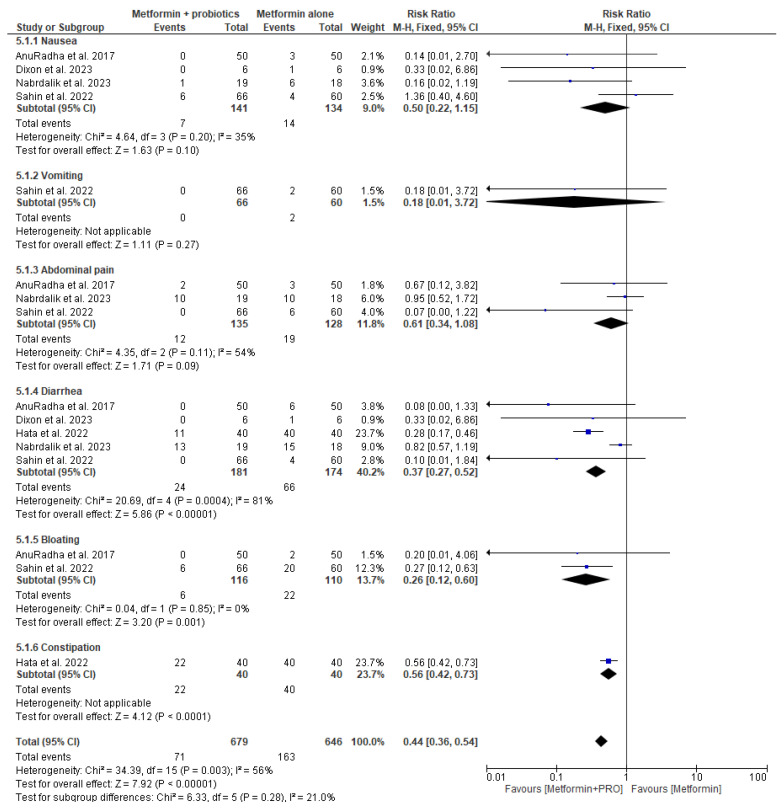
Risk ratio for GI adverse events in patients taking metformin + probiotics (intervention) vs. metformin monotherapy (comparator) [33,36,41,46,53].

We determined whether adding probiotics to metformin therapy may reduce the risk of GI adverse events, as presented in Figure 7. For metformin and probiotics (intervention) vs. metformin alone (comparator), five original papers were eligible for the meta-analysis. Pooled data included 679 patients in the metformin and probiotics intervention group and 646 patients in the metformin alone group. In the analysis in subgroups based on the type of GI adverse events, we observed a pronouncedly lowered risk of diarrhea (RR = 0.37; 95% confidence interval [CI]: [0.27, 0.52], *p* < 0.00001), bloating (RR = 0.26; 95% confidence interval [CI]: [0.12, 0.60], *p* < 0.001), and constipation (RR = 0.56; 95% confidence interval [CI]: [0.42, 0.73], *p* < 0.0001) in the group of patients taking metformin and probiotics compared to group of patients taking metformin alone. We did not find any significant risks of nausea (RR = 0.50; 95% confidence interval [CI]: [0.22, 1.15], *p* = 0.10), vomiting (RR = 0.18; 95% confidence interval [CI]: [0.01, 3.72], *p* = 0.27), or abdominal pain (RR = 0.61; 95% confidence interval [CI]: [0.34, 1.08], *p* = 0.09) in the intervention group vs. comparator group. Therefore, taking probiotics may be beneficial for patients treated with metformin because it reduces the risk of diarrhea, bloating, and constipation.

## 3. Discussion

Metformin is widely used for the treatment of T2DM, However, up to 20% of patients’ experience GI side effects, which significantly affect diabetes control or may be the factor contributing to discontinuation of therapy. Thus, the purpose of our meta-analysis was to assess whether adding other GLDs or probiotics to metformin monotherapy affects the risk of GI side effects.

As expected, our results have confirmed that metformin treatment markedly elevates the risk of abdominal pain, nausea, and vomiting as compared to placebo. Our result are in agreement with previous data that indicated higher risks of abdominal pain and nausea as compared to placebo [11]. Fujioka et al. have observed more incidents of nausea and vomiting in patients treated with metformin as compared to placebo [37].

We did not identify any marked risks of GI side effects between MXR and MIR treatment (Figure 2). Contrarily, increased risks of bloating and diarrhea have been identified for MIR compared to MXR [11]. Tan et al., Aiken et al., and Tarry-Adkins et al. also conducted meta-analyses that compared different forms of metformin in terms of the risk of GI side effects [58,59,60]. Tan et al. found no differences in safety between MXR and MIR [58]. In turn, Tarry-Adkins et al. and Aiken et al. observed a significant reduction in GI side effects in patients taking delayed-release formulation of metformin (MDR) compared to MIR [59,60]. Derosa et al. have observed a lower incidence of diarrhea in patients taking MXR as compared to MIR [35]. In turn, Schwartz et al. have identified lower incidence of nausea in patients taking MXR as compared to MIR [55].

The results of our meta-analysis indicate that metformin monotherapy was associated with higher risks of diarrhea and abdominal pain and a lower risk of vomiting and bloating than other GLDs. In the line with our results, other authors have shown increased risks of abdominal pain, nausea, and diarrhea in patients taking metformin than other antidiabetic drugs. Interestingly, the increased risk of bloating was only observed when metformin was compared to dipeptidyl peptidase-4 inhibitors, DPP4i [11]. The meta-analysis conducted by Wu et. has identified that dipeptidyl peptidase-4 inhibitors are related to a lower incidence of GI adverse events as compared to metformin as well as glucagon-like peptide 1 receptor agonists, metformin, and α-glucosidase inhibitor [61]. In turn, Sun et al. performed a meta-analysis that showed a marked increase in the incidence of GI adverse events for glucagon-like peptide-1 receptor agonists compared with placebo or conventional treatment such as treatment with metformin, thioglitazones, sulfonylureas, insulin, and sitagliptin [62]. Oh et al. have observed higher incidences of diarrhea but lower incidences of vomiting, in patients treated with metformin as compared to other GLDs [49]. In turn, Reasner et al. have revealed more incidence of diarrhea and abdominal pain in patients taking metformin than other GLDs [51]. Additionally, Nauck et al. have identified lower incidence of nausea in patients treated with metformin as compared to those treated with other GLDs [48].

Our meta-analysis has revealed that adding other GLDs to metformin therapy was connected with an elevated risk of nausea and vomiting in comparison to metformin monotherapy. Contrarily, the results obtained by Nabrdalik et al. have suggested that that adding other GLDs to metformin therapy was not related to the increased risk of GI adverse events as compared to metformin monotherapy [11]. Similarly, to our observations, Ratner et al. have observed higher incidences of nausea and vomiting in patients treated with metformin and other GLDs compared to those in metformin monotherapy [50].

The literature data indicate that probiotics may prevent the occurrence of GI disorders after certain medications [63,64,65]. Therefore, we decided to assess whether this beneficial effect also occurs in patients treated with metformin. Since combined therapy of metformin with other GLDs is connected with GI adverse events, our final analysis aimed to check whether co-administering probiotics with metformin treatment influenced the therapeutic risks. We found a decreased risk of diarrhea, bloating, and constipation for metformin and probiotics in relation to metformin monotherapy. To the best of our knowledge, this is the first meta-analysis to explore the effect of addition of probiotics to metformin therapy on the risk of GI adverse events, although a few clinical trials have been conducted that focused on this issue. Firstly, Hata et al. observed a lower incidence of diarrhea and constipation in patients taking metformin with probiotics than that in patients taking metformin monotherapy [41]. In turn, Sahin et al. showed a lower incidence of bloating in patients treated with metformin and probiotics as compared to those treated only with metformin [53]. Another authors have revealed a lower incidence of nausea, abdominal pain, and diarrhea in patients treated with metformin and probiotic compared to that in patients treated with metformin alone [46]. Similarly, Dixon et al. observed a lower incidence of nausea and diarrhea in patients taking metformin with probiotics compared to that in patients taking metformin monotherapy [36]. AnuRadka identified a lower incidence of nausea, abdominal pain, and bloating in patients treated with metformin and probiotics compared to that in patients treated with only metformin [33]. However, further randomized clinical trials involving a larger number of participants should be performed to confirm the beneficial action of probiotics in metformin users.

Our meta-analysis has a few limitations. Firstly, due to the lack of data, we are unable to perform a meta-analysis that will compare different forms of metformin such as MXR, MIR, and MDR with placebo, other GLDs, combinations of metformin with other GLDs, and combinations of metformin with probiotics. Secondly, many studies were excluded from the analysis due to the lack of data on side effects, which limited the amount of data analyzed. Thirdly, no information or data are available on other factors such as treatment with drugs that inhibit the organic cation transporter 1 (e.g., proton pump inhibitors, tricyclic antidepressants, clopidogrel, etc.) that have or may affect the tolerance of metformin [19]. Fourthly, variations in probiotic strains and dosages of probiotics, metformin, and other GLDS could influence the outcomes. The studies included in this meta-analysis used different strains of bacteria (*Bifidobacterium Bifidum G9-1*, *Lactobacillus sporagnes*, *Streptococcus fecalis*, *Clostridium butyricum*, *Bacillus messentericus*, Multistrain probiotic: *Bifidobacte-rium bifidum W23*, *Bifidobacterium lactis W51*, *Bifidobacterium lactis W52*, *Lactobacillus acidophilus W37*, *Levilactobacillus brevis W63*, *Lacticaseibacillus casei W56*, *Ligilactobacillus salivarius W24*, *Lactococcus lactis W19*, and *Lactococcus lactis W58*), and the supplementation times (i.e., month, 12 weeks, etc.) between individual studies were also different. It is also worth emphasizing the importance of the existing intestinal dysbiosis in patients with T2DM, or even small intestinal bacterial overgrowth (SIBO) disease, which significantly influences the frequency of GI adverse events, and this was not taken into account in the studies included in the meta-analysis. Finally, it is worth noting that a very important determinant is the patient compliance with medical recommendations, which may significantly affect the obtained results.

In summary, the obtained results support the addition of probiotics to metformin therapy in patients with T2DM. Nevertheless, there is a need for more well-planned, randomized clinical trials, and the results of which will provide more information regarding the effectiveness of the type of probiotic used in terms of composition and duration of supplementation. There are many factors that influence the incidence of GI adverse events in patients treated with metformin. Among these factors, we highlight the following: dose, metformin formulation (MXR, MIR), frequency of intake per day, and time of metformin intake before a meal (60 min or 30 min before), during a meal, or just after eating. It is of particularly importance, especially as the latest research has revealed that the administration of metformin 60 or 30 min before a meal is not associated with a significantly higher incidence of GI side effects compared to its administration with a meal. Furthermore, this pattern of metformin intake was found to reduce to a greater extent postprandial glycemia and increases the release of GLP-1 [66]. However, these desired findings derived from randomized crossover clinical trial involving 16 participants require further evaluation on a larger group of patients with T2DM.

## 4. Materials and Methods

### 4.1. Search Strategy

PubMed/Medline, Web of Science, Embase, Cochrane Central Register of Controlled Trials, and ClinicalTrials.gov databases were thoroughly searched from December 2023 to April 2024. Moreover, the analysis of gray literature (non-peer-reviewed publications, conference proceedings, patents, published reports or datasets, and whitepapers) from Google Scholar was also conducted. The searching strategy was as follows: PubMed/Medline, Cochrane Central Register of Controlled Trials—((insulin resistance) OR (diabetes)) AND ((metformin) OR (biguanide)) AND ((treatment) OR (therapy)).

### 4.2. Selection Criteria

Based on the Population, Intervention, Comparison, Outcomes, and Type of Study (PICOT) structure, selection criteria and research questions were formulated. The inclusion criteria comprised the following: (P)—patients with T2DM; (I)—metformin; (C)—placebo or metformin and other GLDs from the group of sulfonylurea derivatives, glitazones, and DPP-IV inhibitors or metformin and probiotics; (O)—adverse events from the GI tract, such as diarrhea, abdominal pain, nausea, vomiting, flatulence, and constipation; and (T)—RCTs. Thus, we included the following types of studies: randomized placebo controlled clinical trials (metformin vs. placebo) and head-to-head trials ([MXR] vs. [MIR], metformin monotherapy vs. metformin and other GLDs, and metformin and probiotics vs. metformin monotherapy). These studies were performed on patients suffering from T2DM treated with metformin in monotherapy or in combination with other GLDs or other GLDs in which GI adverse events were reported. The management of hyperglycemia in T2DM followed the current recommendations. The management was focused on glycemic treatment targets, mainly HbA1c level, age, risk of developing diabetes complications, and the risk of adverse effects of therapy (e.g., hypoglycemia and weight gain).

The exclusion criteria included the following: (1) review articles, (2) meta-analysis, (3) case reports, (4) case series, (5) cohort studies, (6) observational studies, (7) conference abstracts, (8) animal research, (9) articles with scarce data, (10) articles with scarce information, (11) articles not published in English, and (12) studies in which it was impossible to extract individual results.

### 4.3. Selection of Studies and Extraction of Data

All selected articles that met the inclusion criteria were downloaded in full-text version for deeper analysis and review. Two independent investigators evaluated each selected study to reduce the risk of potential selection bias. These two investigators also made the final decision regarding the inclusion or exclusion of a given study, which was in accordance with the specified inclusion criteria. In order to identify duplicate reports from the same study, the steps taken were as follows: (1) the date and duration of the study were compared, (2) the names of the authors of the study were compared, (3) if there was more than one article that had one or more authors in common, the identification numbers of studies were checked (if applicable), (4) repeated names of institutions were checked, (5) intervention details were compared, and (6) the number of study participants and results were compared. The selection was made by two independently working researchers. Finally, as a result of the selection process, duplicates were rejected. One researcher extracted the data, and the other researcher reviewed the data. In the case of inconsistency or lack of agreement between researchers, negotiations were conducted until consensus was reached. The following information was separated from the studies included in the analysis: title, authors, number and age of subjects, exposure, outcome, and number of cases. The study investigator reviewed titles and abstracts. Then, a full-text review was conducted to confirm that both inclusion and exclusion criteria were met.

### 4.4. Evaluation of the Risk of Bias and Quality of Methodology

The Cochrane Review Manager 5.4 software tool was employed by two independent researchers to evaluate the methodological quality. In the case of differing opinions, the final decision was made based on the discussion process. The allowable value of losses that may affect the test results was set at 10%. Studies were segregated into the following classifications: low risk of bias, unclear risk of bias, and high risk of bias.

### 4.5. Statistical Analysis

A standardized database was employed to collect extracted data. Then, the data were analyzed using the Cochrane Review Manager 5.4 software. We performed several subgroup analyses stratified by study design based on the type of intervention. Subgroup analyses were presented using a forest plot. The final results included dichotomous data represented as (RR) and 95% confidence interval (CI) in each group. I^2^ evaluated heterogeneity between results of studies. The I^2^ value was interpreted as follows: 0–30%, low level of heterogeneity; 30–60%, moderate level of heterogeneity; and over 60%, high level of heterogeneity. When *p* < 0.05, then, the results of this meta- analysis were considered as statistically significant.

## 5. Conclusions

Our study showed that the administration of probiotics together with metformin was associated with a reduced risk of diarrhea, bloating, and constipation. Thus, taking probiotics may have potential benefits for patients who experience GI side effects during treatment with metformin. Therefore, adding probiotics to metformin therapy should be considered for patients experiencing GI side effects associated with the drug. This meta-analysis was registered in PROSPERO, ID: CRD42024535353.

## Data Availability

Data is contained within the article.
